# Data on media use and mental health during the outbreak of COVID-19 in China

**DOI:** 10.1016/j.dib.2021.106765

**Published:** 2021-01-19

**Authors:** Dini Xue, Tour Liu, Xueming Chen, Xiaorui Liu, Miao Chao

**Affiliations:** a Key Research Base of Humanities and Social Sciences of the Ministry of Education, Academy of Psychology and Behavior, Tianjin Normal University, Tianjin, China; b Faculty of Psychology, Tianjin Normal University, Tianjin, China; c Center of Collaborative Innovation for Assessment and Promotion of Mental Health, Tianjin Normal University, Tianjin, China

**Keywords:** COVID-19, Media use, Boredom, Empathy and sympathy, Positive and negative affect, Death anxiety, Meaning in life, Depression, Anxiety, Stress

## Abstract

The Coronavirus disease (COVID-19) spread rapidly in China in beginning of 2020. Self-quarantine was suggested by Chinese government to block the spread of the COVID-19. During the self-quarantine, the media played an indispensable role in acquisition of information about the disease. And it could also impact on people's mental health. Therefore, it is necessary to study the psychological outcome resulted from media use during the outbreak of COVID-19. The data in this article could help researchers to explore the mechanism between media use and mental health, and to have a deeper comprehension of the impact of media use on mental health during a public health emergency. The dataset provided in this article included 917 participants recruited from different provinces all over China. Among the participants, there were 304 males and 613 females, with an average age of 28.6 and a standard deviation of 9.5. They took Media Use Questionnaire (MUQ), Empathy-Sympathy Questionnaire (ESQ), Positive and Negative Affect Scale (PANAS), Death Anxiety Questionnaire (DAQ), Meaning in Life Questionnaire (MLQ), State boredom Questionnaire (SBQ), and Depression Anxiety Stress Scale-21 (DASS-21) to assess their media use and mental health during the outbreak of COVID-19. All these instruments for data collection were Chinese versions. In addition, a .csv file consists of major variables we used are included as a supplementary material. Descriptive statistics and hierarchical regression had been conducted with these data. For a discussion of the findings based on the dataset please see the article: Media use and acute psychological outcomes during COVID-19 outbreak in China [1] and Psychological distress and state boredom during the COVID-19 outbreak in China: the role of meaning in life and media use [2].

## Specifications Table

**Table:** 

Subject	Psychology
Specific subject area	Psychological assessment of social psychology
Type of data	Microsoft Excel Comma Separated value document(.csv), Table, Figure
How data were acquired	Questionnaires
Data format	Raw, Analyzed
Parameters for data collection	Background: During the COVID-19 outbreak in China Variables: Media use, Boredom, Empathy and Sympathy, Positive and negative affect, Death anxiety, Meaning in Life, Depression, Anxiety, Stress
Description of data collection	We conducted an investigation on January 28, one week after the National Health Commission of China announced to the public that COVID-19 could be transmitted interpersonally. All participants were informed of the study purpose and provided consent to participate. The survey uses a Tencent questionnaire to conduct online surveys and avoid multiple participation by recording the device's IP address. The research questionnaire link was originally shared by several teachers and students of Tianjin Normal University through WeChat Moments (a popular Chinese social media platform). Share the link to more people through the snowball sampling method.
Data source location	China
Data accessibility	Data we used were provided as supplementary material. https://github.com/liutour/Media-Use-and-Mental-Health-during-COVID19
Related research article	Chao, M., Xue, D., Liu, T., Yang, H., & Hall, B. J., 2020. Media use and acute psychological outcomes during COVID-19 outbreak in China. J. Anxiety Disorders. 74, 102248. https://doi.org/10.1016/j.janxdis.2020.102248. Chao, M., Chen, X., Liu, T., Yang, H., & Hall, B. J., 2020. Psychological distress and state boredom during the COVID-19 outbreak in China: the role of meaning in life and media use. European J. Psychotraumatology. 11, 1769379. http://doi.org/10.1080/20008198.2020.1769379.

## Value of the Data

• The dataset provided some important information about media use and mental health during the outbreak of COVID-19 in China.
• These data could help researchers to understand and explore the relationships between people's mental health and media use during the outbreak.
• These data can be used in the Structural Equation Model (SEM) and other analyses.
• These data were measured in the background of Chinese culture and researchers can compare the differences in people's psychological status during the disease in different cultural backgrounds and that may promote multicultural communication.
• These data set were conducive to exploring the impact of media use on public mental health under major public health events similar to this outbreak.

## 1 Data Description

The .csv file presented the data concerning people's media use (media content, new media and traditional media), boredom, empathy, sympathy, positive and negative affect, death anxiety, meaning in life, depression, anxiety and stress during the outbreak of COVID-19 in China. The data was collected from an online platform "Tencent Questionnaire” from January 28 to January 29, 2020 (one week after the official declaration of person-to-person transmission of the COVID-19). The questionnaire link was initially shared via WeChat (the most popular Chinese social media platform) by a dozen teachers and students from Tianjin Normal University. And people who finished the questionnaire were encouraged to share the link with more people via a snowball sampling method. Except for the locations of participants, the dataset did not include any missing values. We have provided the English-version of the questionnaire in the supplementary files. For a further discussion of the major finding based on this dataset please see the article: Media use and acute psychological outcomes during COVID-19 outbreak in China (https://doi.org/10.1016/j.janxdis.2020.102248) [1] and Psychological distress and state boredom during the COVID-19 outbreak in China: the role of meaning in life and media use (http://doi.org/10.1080/20008198.2020.1769379) [2]. Part of the questionnaire answers were presented in [2].

- the Table 1 shows the descriptive statistics of the media use variables and the people's mental health variables.

**Table 1:** Descriptive statistic results of media use and mental health variables.

Variables	N	Minimum	Maximum	Mean	SD
1.Media content	917	6.00	30.00	22.30	3.93
2.New media	917	0.00	47.00	12.53	10.71
3.Traditional media	917	0.00	12.00	3.04	3.23
4.Boredom	917	3.00	21.00	11.72	5.48
5.Positive affect	917	10.00	50.00	28.59	6.02
6.Negative affect	917	10.00	50.00	22.91	7.42
7.Death anxiety	917	4.00	20.00	12.10	3.78
8.Meaning in life	917	4.00	28.00	21.43	4.39
9.Empathy	917	2.00	10.00	6.53	2.12
10.Smpathy	917	2.00	10.00	8.25	1.44
11.Depression	917	7.00	26.00	10.77	3.27
12.Anxiety	917	7.00	26.00	11.07	3.01
13.Stress	917	7.00	26.00	12.32	3.45
14. Posting information	917	0.00	1.00	0.48	0.50
15.Actively searching	917	1.00	4.00	3.01	1.04
- the Table 2 shows the descriptive statistics of variables for living situation and attitude toward COVID-19.

**Table 2:** Descriptive statistics of variables for the living situation and attitude toward epidemic.

Variables	N	Minimum	Maximum	Mean	SD
The number of family members	917	1.00	11.00	3.91	3.30
Is there anyone around you has been infected	917	0.00	0.00	0.00	0.00
How much impact does the epidemic have on your life?	917	1.00	5.00	3.29	0.83
Family reunion	917	0.00	1.00	0.90	0.30
Family relationship	917	1.00	5.00	4.32	0.79
Are you optimistic about the epidemic?	917	1.00	5.00	2.88	1.07

## 2 Experimental Design, Materials and Methods

### 2.1 Participants

**Fig. 1:**
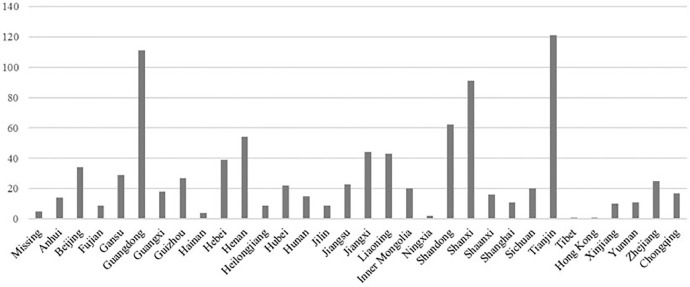
The specific locations of the participants.

The data presented in this article were collected from 917 participants (we first recruited a total of 932 participants and deleted the cases whose friends or families were infected by coronavirus) during the outbreak of COVID-19 in China. Among them, there were 304 males and 613 females, with an average age of 28.6 and a standard deviation of 9.5. The locations of participants were shown in Fig. 1 [3]. In this data set, some demographic information such as age, gender, marital status, educational level and so on were deleted for confidentiality.

### 2.2 Questionnaires

#### 2.2.1 Media Use Questionnaire (MUQ)

Media use was measured by a 11-item Media Use Questionnaire (Chinese version). This questionnaire was specially developed in the outbreak context to measure the level of an individual's involvement in media information about the COVID-19. This questionnaire involved two dimensions: media content (6 items) and media use time (4 items for new media and 1 item for traditional media). For the media use time dimension, participants indicated the number of total hours in the last week that they were exposed to the coverage of the disease outbreak. An example was “In the past week, how long did you spend watching TV programs about the epidemic, listening to the radio about the epidemic, and reading the newspaper about the epidemic?” Media content items were measured on 5-point scale (1 = never, 2 = rarely, 3 = sometimes, 4 = often, 5 = always). An example was, “How often do you view content on the severity of the outbreak nationally and regionally?” A higher score indicates a higher level of media involvement. In addition, participants had responded to another two items which were also used to assess media involvement. They are “Whether they had shared the epidemic-related information on social media” and “How often do you actively search for news updates on the epidemic in the last week”.

#### 2.2.2 State boredom

Boredom was measured by State boredom Questionnaire (Chinese version). The questionnaire is to measure state boredom during the outbreak and contains three items, including ‘During the last week, I often find myself at loose ends, not knowing what to do’; ‘During the last week, many things I have to do are repetitive and monotonous’; and ‘During the last week, I just sit around doing nothing’. Items are scored on 1-7 (1 = totally disagree, 2 = more disagree, 3 = slightly disagree, 4 = unsure, 5 = slightly agree, 6 = more agree, 7 = fully agree). A higher score indicates higher state boredom. Exploratory factor analysis supports unidimensionality. The Cronbach’ α was 0.86.

#### 2.2.3 Empathy-Sympathy Questionnaire (ESQ)

Empathy and sympathy were measured by a 4-item Empathy-Sympathy Questionnaire (Chinese version). This questionnaire was revised in the outbreak context to reflect people's empathy and sympathy in the COVID-19 outbreak, and the original questionnaires were Renate's version of empathy scale [4] and Sherman's version of sympathy scale [5]. The questionnaire consisted of two subscales: empathy (2 items) and sympathy (2 items). Items were measured on a 5-point Likert scale (1 = totally disagree, 2 = disagree, 3 = uncertain, 4 = agree, 5 = totally agree) for each subscale. An example question was, “If other people feel nervous and worried due to the outbreak, I would feel nervous and worried too”. A higher score indicates a higher level of individual empathy or sympathy. The internal consistency coefficient (α) was 0.64. Exploratory factor analysis displayed the two factors structure of the questionnaire, the factor load of each item was between 0.74 and 0.92.

#### 2.2.4 Positive and Negative Affect Scale (PANAS)

Positive and Negative affect was measured by Positive and Negative Affect Scale (Chinese version) which was developed by Watson [6]. The scale involved two dimensions: positive affect (10 items) and negative affect (10 items). Items were rated on a 5-point Likert scale (1 = almost none, 2 = less, 3 = intermediate, 4 = more, 5 = extremely much). A higher positive affect score indicates that the individual's energetic, concentrate and happy emotional state, while a lower score indicates indifference. A higher negative affect score indicates an individual's subjective feelings of confusion and pain, and a lower score indicates calmness. The scale showed good reliability and validity [7]. In the present study, the internal consistency coefficient (α) of positive and negative affect were 0.84 and 0.90 respectively.

#### 2.2.5 Death Anxiety Questionnaire (DAQ)

Death anxiety was measured by a Chinese version of Death Anxiety Questionnaire which was revised from Donald's version [8]. This 4-itme scale could reflect people's death fear and worry related to the COVID-19 outbreak. Items were measured on a 5-point Likert scale (1 = totally disagree, 2 = disagree, 3 = uncertain, 4 = more agree, 5 = totally agree). An example was, “The topic of coronavirus causing illness and death makes me feel distressed.” A higher score indicates a higher degree of worry and fear of death. The internal consistency coefficient (α) of the questionnaire was 0.78, the results of the exploratory factor analysis supported the unidimensionality of the questionnaire.

#### 2.2.6 Meaning in Life Questionnaire (MLQ)

A 4-item Meaning in Life Scale was adapted from Steger's scale [9] according to the epidemic background. It could reflect the degree of satisfaction with life during the COVID-19 outbreak. Items were measured on a 7-point Likert scale (1 = absolutely untrue, 2 = mostly untrue, 3 = somewhat untrue, 4 = can't say true or false, 5 = somewaht true, 6=mostly true, 7=absolutely true). An example question was, “My life is close to my ideal in most respects”. The questionnaire showed good reliability and validity, the internal consistency of the scale was 0.85.

#### 2.2.7 Depression Anxiety Stress Scale-21 (DASS-21)

The Chinese version of Depression Anxiety Stress Scale-21 (DASS-21) was used in the current study [10]. This scale was revised from Antony's scale [11]. The scale could reflect people's depression, anxiety and stress during the COVID-19 outbreak. This scale involved three dimensions: depression (7 items), anxiety (7 items) and stress (7 items). Items were measured on a 5-point scale (1 = never, 2 = rarely, 3 = sometimes, 4 = often, 5 = always). The DASS-21 has good reliability and validity, the internal consistency of the three subscales was between 0.76 and 0.79, and the internal consistency of the total scale was 0.89.

### 2.3 Statistical analysis

The results of descriptive statistics (*Mean* and *SD*) and correlations among the total scores of major variables in the questionnaires are presented in Table 1 and Table 2.

## Ethics Statement

This study was approved by the ethics committee of Tianjin University (XL2020-12). The online questionnaire was anonymous, and all participants were informed of the study purpose and provided consent.

## Declaration of Competing Interest

The authors declare no competing interests.

## Supplementary Material

Supplementary materials to this article can be found online at https://github.com/liutour/Media-Use-and-Mental-Health-during-COVID19.
